# Smoking Cessation Intervention for Severe Mental Ill Health Trial (SCIMITAR+): study protocol for a randomised controlled trial

**DOI:** 10.1186/s13063-017-1789-7

**Published:** 2017-01-26

**Authors:** Emily Peckham, Catherine Arundel, Della Bailey, Stuart Brownings, Caroline Fairhurst, Paul Heron, Jinshuo Li, Steve Parrott, Simon Gilbody

**Affiliations:** 1 Mental Health and Addiction Research Group, University of York, Heslington, YO10 5DD UK; 2 York Trials Unit, University of York, Heslington, YO10 5DD UK; 3 Department of Psychiatry, University College London, Gower Street, London, UK; 4 Kent and Medway NHS and Social Care Partnership Trust, Farm Villa, Hermitage Lane, Maidstone, London, UK

**Keywords:** Smoking cessation, Nicotine replacement therapy, Complex intervention, Schizophrenia, Bipolar disorder, Severe mental ill health, Randomised controlled trial

## Abstract

**Background:**

Smoking is highly prevalent among people who have experience of severe mental ill health, contributing to their poor physical health. Despite the ‘culture’ of smoking in mental health services, people with severe mental ill health often express a desire to quit smoking; however, the services currently available to aid quitting are those which are widely available to the general population and may not be suitable or effective for people with severe mental ill health. The aim of this study is to explore the effectiveness and cost-effectiveness of a bespoke smoking-cessation intervention specifically targeted at people with severe mental ill health.

**Methods/design:**

SCIMITAR+ is a multicentre, pragmatic, two-arm, parallel-group, individually randomised controlled trial.

We aim to recruit 400 participants aged 18 years and above with a documented diagnosis of bipolar disorder, schizophrenia or schizoaffective disorder who smoke. Potentially eligible participants identified in primary or secondary care will be screened, and baseline data collected. Eligible, consenting participants will be randomly allocated to one of two groups. In the intervention arm, the participant will be assigned a mental health professional trained to deliver smoking-cessation interventions who will work with the participant and participant’s GP or mental health specialist to provide an individually tailored smoking-cessation service. The comparator arm will be usual care – following current NICE guidelines for smoking cessation, in line with general guidance that is offered to all smokers, with no specific adaptation or enhancement in relation to severe mental ill health.

The primary outcome will be self-reported smoking cessation at 12 months verified by expired carbon monoxide (CO) measurement. Secondary outcome measures include Body Mass Index at 12 months, the Fagerström Test for Nicotine Dependence, Motivation to Quit questionnaire, SF-12, PHQ-9, GAD-7, EQ-5D-5 L, and health service utilisation at 6 and 12 months. The economic evaluation at 12 months will be conducted in the form of an incremental cost-effectiveness analysis.

**Discussion:**

SCIMITAR+ trial is the largest trial to our knowledge to investigate the effectiveness of a bespoke smoking-cessation service for people with severe mental ill health.

**Trial registration:**

International Standard Randomised Controlled Trials Number, ISRCTN72955454. Registered on 16 January 2015.

**Electronic supplementary material:**

The online version of this article (doi:10.1186/s13063-017-1789-7) contains supplementary material, which is available to authorized users.

## Background

The physical health of people with severe mental ill health (SMI), such as schizophrenia and bipolar disorder, is often poor with people with a diagnosis of schizophrenia reported to die 20–25 years earlier than those in the general population [1]. One of the largest contributory factors to this early mortality is smoking [2]. Smoking is a preventable health hazard, with proven associations with diseases such as cancer and heart disease. A recent study compared outcomes for people with SMI who smoke to people with SMI who do not smoke and found that on average people with SMI who smoke die nearly 10 years earlier than those who do not smoke [3]. Smoking is, therefore, one of the most important modifiable risk factors for this excess mortality, though smoking may be only one of several reasons for this observed difference. However, whilst the number of people in the general population who smoke has been steadily declining over recent decades [4], the number of people with SMI who smoke has not been declining at the same rate. People with SMI are still more likely to smoke [5] and to smoke more heavily, extracting more nicotine from each cigarette, than those in the general population [6].

Estimates of the percentage of people with SMI who smoke vary depending on the setting with up to 70% of inpatients smoking [7, 8]. Despite this, the percentage of people with SMI who, when asked, express a desire to cut down or quit smoking is not dissimilar to that in the general population when asked the same question [9]. However, although people with SMI express a desire to quit smoking they are less likely to receive help in quitting compared with the general population [10, 11]. This is in part due to the culture of smoking within mental health services amongst staff and patients [12, 13] where cigarettes in the past have been used as a currency between service users. There are widely held myths about the therapeutic function of smoking and that smoking relieves anxiety when in fact nicotine can increase anxiety [14]. In addition, smoking is perceived by some to help alleviate depression whereas a systematic review has found smoking cessation to reduce depression [15]. Furthermore, conventional NHS approaches to smoking cessation do not take into account the additional challenges that people with SMI may face when attempting to quit smoking. Conventional services place a strong emphasis on being able to set a quit date from the outset which may deter people with SMI. In addition, smoking-cessation services for people with SMI are not yet sufficiently evolved or embedded within the NHS.

In order to address these problems, we have developed a bespoke smoking-cessation intervention specifically tailored to people with SMI. We initially piloted this intervention in the Smoking Cessation Intervention for severe Mental Ill health Trial (SCIMITAR) to assess its acceptability and uptake by people with SMI [16]. In our bespoke smoking-cessation intervention, a mental health nurse or allied health professional is trained to deliver smoking-cessation interventions and to work with the individual and their GP or Community Mental Health Team (CMHT). They advise on antismoking medication and provide behavioural support in the form of information, encouragement, and motivational sessions on cutting down to quitting, setting quit dates and maintaining smoking abstinence. The service is similar to that offered in regular smoking-cessation services, but with specific adaptations of medication and tailored behavioural support to the individual needs of people with SMI. The SCIMITAR pilot trial found the intervention to be acceptable to people with SMI and that it was feasible to recruit and retain participants, randomising 97 participants to receive either the bespoke smoking-cessation service or usual care [16]. The clinical and cost-effectiveness of this bespoke smoking-cessation service will now be evaluated in the definitive randomised controlled SCIMITAR+ trial.

### Research objectives

1. To establish the clinical effectiveness of a bespoke smoking-cessation intervention compared with usual care for people with severe mental ill health.
2. To establish the cost-effectiveness of a bespoke smoking-cessation intervention for people with severe mental ill health.

## Methods/design

**Fig. 1:**
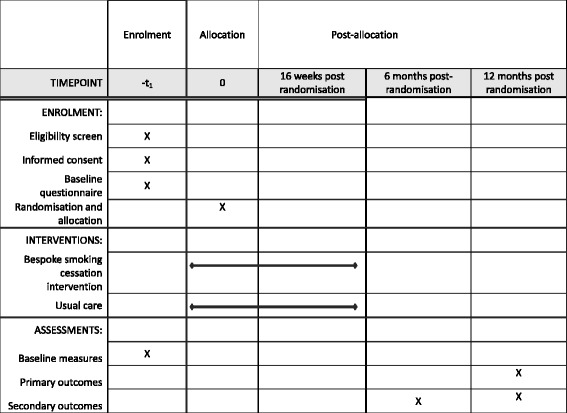
Standard Protocol Items: Recommendations for Interventional Trials (SPIRIT) figure – schedule of enrolment, intervention and assessments

The SCIMITAR+ study is a two-arm, pragmatic, parallel-group, randomised controlled trial. It is a multicentre study (22 sites in the UK) recruiting from both specialist mental health services and primary care and aims to recruit a sample size of 400 participants. Ethical approval for this study was sought and received from Leeds East Research Ethics Committee on 19 March 2015 (REC Ref: 15/YH/0051). The schedule of enrolment, interventions and assessments is shown in Fig. 1. Additional file 1: Table S1 presents the Standard Protocol Items: Recommendations for Interventional Trials (SPIRIT) Checklist.

### Identification and recruitment

Potential participants will be recruited using five methods:

1. *GP database screening*
  Participating GP practices will search their patient databases and SMI register to identify potential participants. Suggested Read codes will be provided to assist with database searches. Participant information packs will be sent to identified patients by the GP practice to invite them to take part in the study. Those interested in taking part will be asked to complete and return a *Consent to be Contacted* Form to the SCIMITAR+ researchers, who will then approach the potential participant to confirm eligibility and obtain consent.
2. *Direct GP referral or primary care referral following annual health check*
  People with SMI consult with their GPs frequently, largely in connection with physical rather than mental health problems. Current guidelines encourage GPs to offer opportunistic advice and information about smoking-cessation services to all patients who smoke whenever they consult in primary care. Such opportunistic advice provides a chance for GPs to introduce the SCIMITAR+ trial to potential participants. Those interested in taking part will be asked to complete a *Consent to be Contacted* Form which the GP will return to the SCIMITAR+ researchers, who will then approach the potential participant to confirm eligibility and obtain consent.
3. *Direct referral via Community Mental Health Teams (CMHTs) and psychiatrists*
  SCIMITAR+ researchers will work with care coordinators and consultant psychiatrists to screen their entire caseloads for potential participants who match the inclusion criteria. Those identified as potentially suitable for the SCIMITAR+ trial will be given a copy of the participant information pack by their care coordinator or psychiatrist. The participant information pack will contain a *Consent to be Contacted* Form which potential participants can return to the SCIMITAR+ research team
  Members of the CMHT will also be invited to directly refer potential participants to the research team, following a similar pathway to GP referrals. Posters and flyers advertising the study will be displayed in centres recruiting to the study, on Facebook and Twitter accounts, and in third-sector community organisations. Interested service users can contact the mental health professional named on the flyer, or a member of their care team, to request participant information packs.
4. *Recruitment from service user groups*
  Service user groups will be provided with information about the study along with copies of a SCIMITAR flyer. Interested service users can contact the mental health professional named on the flyer or their care coordinator. Alternatively, the staff member at the service user group can contact the service user’s care coordinator on their behalf.
5. *Recruitment via the Lifestyle Health and Wellbeing Survey*
  The Lifestyle Health and Wellbeing Survey is a questionnaire-based study, coordinated by the University of York (REC ref: 15/WM/0444) involving adults aged 18 years and over with SMI recruiting from direct referrals of primary and secondary care teams. Participants in the Lifestyle Health and Wellbeing Survey complete a series of questions about their health and wellbeing including questions about their smoking status. Survey respondents who smoke and are potentially interested in cutting down or quitting smoking, and have consented to being contacted by the research team, will be invited to take part in SCIMITAR+.

### Screening for eligibility

Once a potential participant has given consent to be contacted (either verbally to the recruiting clinician or in writing), a SCIMITAR+ researcher will approach them either by telephone, face to face or by e-mail depending on the participant’s preference. After briefly explaining the trial the researcher will check the participant’s eligibility and then, if eligible, will arrange a face-to-face appointment at a mutually convenient time and venue.

At the face-to-face appointment the potential participant will be provided with the opportunity to ask any additional questions about the study and provide written informed consent. Once written consent has been obtained the participant will complete the baseline questionnaire with the researcher.

### Inclusion and exclusion criteria

Inclusion:

- Aged 18 years or over.
- A documented diagnosis of schizophrenia or delusional/psychotic illness (*International Classification of Diseases, version 10* (ICD-10) F20.X and F22.X or *Diagnostic and Statistical Manual of Mental Disorders* (DSM)-equivalent) or bipolar disorder (ICD-10 F31.X or DSM-equivalent).
- Current smoker, smoking at least five tobacco cigarettes per day.
- Express a desire to cut down or quit smoking.

Exclusion:

- Pregnant or breast-feeding women.
- Known comorbid drug or alcohol dependency (as recorded in GP or psychiatric records).
- Currently receiving advice from a stop smoking advisor.
- Non-English speaking.
- Lack of capacity to consent.

### Randomisation and blinding

Eligible, consenting participants will be randomised 1:1 to either a bespoke smoking-cessation service or usual care. The researcher will contact a secure telephone randomisation service run by the York Trials Unit. Simple randomisation will be used, following a computer-generated random number sequence. The researcher will immediately inform the participant of their allocation and what will happen next. A letter will be sent to the GP and mental health specialist for filing in the participant’s records and to advise them on subsequent smoking-cessation management.

Neither the nature of the interventions in this study nor the study design allows for the masking of the therapists or the participants. However, statistical analysis will be conducted blind to treatment allocation.

### Interventions

#### Smoking-cessation intervention

This is a complex intervention involving a bespoke smoking-cessation service provided by a mental health professional trained in smoking-cessation interventions. The mental health smoking-cessation practitioner (MH-SCP) will work in conjunction with the participant and the participant’s general practitioner (GP) or mental health specialist to provide a smoking-cessation service individually tailored to the participant’s needs. The participant will be offered between eight and 12 sessions with the MH-SCP. The intervention will be delivered according to the National Centre for Smoking Cessation and Training’s guidelines with the following specific adaptations for people with SMI: in line with National Institute of Health and Care Excellence (NICE) guidelines for smoking cessation [17]:

1. The possibility of providing several sessions prior to setting a ‘quit date’.
2. Recognising the purpose of smoking in the context of their mental illness, such as the use of smoking to relieve side effects from antipsychotic medication (and how this will be managed during a cessation attempt).
3. The need to involve other members of the multidisciplinary team in planning a successful quit attempt for those with complex care needs and multiagency programmes of care.
4. A greater need for home visits, rather than planned visits in GP surgeries (for some clients with mental illness, the home environment is the optimum place to work).
5. Providing additional face-to-face support following an unsuccessful quit attempt or relapse.
6. Informing the GP and psychiatrist of a successful quit attempt so that they can review antipsychotic medication doses if the participant’s metabolism changes.

### Control group

This will be a ‘usual care’ control group whereby the participants will be signposted towards usual care for their area. Usual care varies according to where the participant is geographically located and is dependent on local commissioning arrangements. One example consists of participants attending their local GP service on a fortnightly basis to have their expired carbon monoxide (CO) measured by the in-house smoking-cessation practitioner and to collect their ongoing nicotine replacement therapy (NRT) prescription. In other areas participants are referred to a local smoke-free NHS service for the same fortnightly meetings but with an additional initial assessment of nicotine dependence and motivation to quit as well as requiring participants to set a ‘quit day’ when they will stop smoking. In some instances, smoke-free services offer weekly individual or group meetings for motivational and behavioural support at a local centre or pharmacy. In another example participants may visit their GP who provides an NRT prescription and refers patients to a local council-funded project for individual behavioural support.

### Outcome measures

Data will be collected from participants at baseline and at 6 and 12 months post randomisation. Baseline and follow-up assessments will take place during face-to-face meetings with the participant in order to measure height and weight to calculate the Body Mass Index (BMI), and to take CO readings. As face-to-face follow-up is preferred every effort will be made to collect, as a minimum, a reduced set of data by this method. Where it is not possible to meet with the participant for face-to-face follow-up data collection, the participant will be offered the option of completing the questionnaires by telephone or have the questionnaires sent to them by post for them to complete and return. If it is not possible to make contact with the participant for follow-up, a family member or friend named by the participant at their baseline interview as a person who could be contacted, will be asked to verify the participant’s smoking status.

The primary outcome measure will be cessation of smoking at 12 months assessed according to the Russell standard. Smoking cessation will be defined as having an expired-air CO measurement of less than 10 ppm *and* self-reporting as a quitter (responding ‘not even a puff’ to the question ‘Have you smoked in the last week?’).

**Fig. 2:**
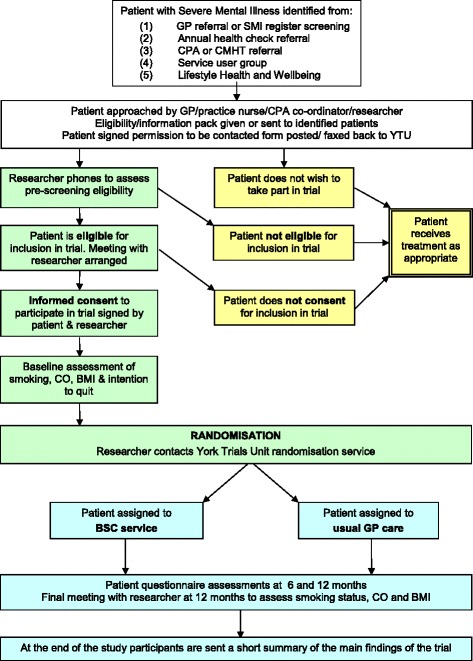
Participant flow through the trial

Secondary outcomes at 6 and 12 months post randomisation will include measures of smoking status, general mental health functioning, BMI, health-related quality of life, health service use, and adherence with smoking-cessation advice from MH-SCP (Table 1). The flow of participants through the trial is shown in Fig. 2.

**Table 1:** Outcome measure and collection time point

Assessment	Time point (months post randomisation)		
	Baseline	6	12
Eligibility and consent			
Eligibility	X		
Consent	X		
Background and follow-up			
Personal details, general health	X		
Body Mass Index	X	X	X
Mental health details			
Mental health history	X		
Current mental health status	X	X	X
Current medications	X	X	X
Referrals to mental health services		X	X
Admissions to hospital related to mental health		X	X
Smoking details			
Smoking history	X		
Current smoking status	X	X	X
Use of electronic cigarettes	X	X	X
Use of smoking-cessation services	X	X	X
CO measurement	X	X	X
Adverse event reporting	Ongoing collection		
Questionnaires			
Fagerström Test for Nicotine Dependence (FTND) [17]	X	X	X
Motivation to Quit questionnaire (MTQ)	X	X	X
Patient Health Questionnaire (PHQ-9)	X	X	X
Generalised Anxiety Disorder Assessment (GAD-7)	X	X	X
Health-related quality of life (SF-12)	X	X	X
Health-state utility (EuroQol – EQ-5D-5L)	X	X	X
Health Economics/Service Utilisation Questionnaire	X	X	X

### Withdrawal

A study participant may be withdrawn from the trial by their general practitioner (GP), mental health specialist, smoking-cessation practitioner or may choose to do so themselves. Reasons for withdrawal may include pregnancy, admission to hospital for reasons unrelated to the trial, inability to attend treatment or assessment sessions. Relapse to resuming smoking is not seen as reason to withdraw since participants can resume treatment and make several attempts to quit smoking. Participants will be able to (1) withdraw from treatment only (participants still followed up at 6 and 12 months) or (2) withdraw fully from the study including from treatment and follow-up. Withdrawal from the study does not affect the patients’ treatment or access to NHS services. Any data collected from the participant prior to withdrawal will still be included in the final analysis of the data. In the case of missing primary outcome data due to withdrawal it will be assumed that the participant is still smoking. For further details about how missing data will be handled please see the ‘Data analysis’ section below.

### Sample size

The SCIMITAR+ trial has been powered to detect a relative increase in quitting of 1.7 (baseline quit rate 20%, intervention quit rate 34%) using a two-sided significance level of 5% at 80% power. This would require a sample size of 314 participants using equal (1:1) randomisation. Allowing for a 20% loss to follow-up would give a total sample size of at least 393. We therefore propose to recruit 400 participants in total to ensure sufficient power.

### Data analysis

All analyses will be conducted on an intention-to-treat basis, including all randomised patients in the groups to which they were allocated where data are available. Analyses will be conducted in STATA v13 or later (StataCorp, College Station, TX, USA). Statistical tests will be two-sided using 5% significance.

The flow of participants through the trial will be described in a Consolidated Standards of Reporting Trials (CONSORT) diagram. The number of withdrawals will be summarised by type, treatment group and time, with reasons where provided.

All participant baseline data will be summarised descriptively by treatment group both as randomised and as analysed in the primary analysis. No formal statistical comparisons will be undertaken.

The primary outcome of CO-verified smoking cessation at 12 months will be analysed via a mixed-effects logistic regression model to compare bespoke smoking-cessation services with usual care. The model will be adjusted for baseline smoking severity (self-reported number of cigarettes smoked) and treatment group, with centre as a random effect. The odds ratio, corresponding two-sided 95% confidence interval and *p* value for the treatment effect will be presented.

In a sensitivity analysis, we will substitute CO-verified results with self-reported cessation results where CO results are missing. Two further sensitivity analyses will be conducted treating participants who still have missing primary outcome data in the following two ways: (1) patients with missing data will be assumed to still be smoking and (2) multiple imputation techniques will be used.

All data collected at 6 and 12 months will be summarised descriptively by treatment group, including an investigation of the level of missing data.

The primary analysis will be repeated using, as the outcome: (1) CO-verified smoking cessation at 6 months, (2) self-reported smoking cessation at 6 months, and (3) self-reported smoking cessation at 12 months.

The number of cigarettes smoked per day will be compared between the two groups using a Poisson regression model, adjusting for the same covariates as the primary analysis. If the variance of the data is larger than the mean, this may give an indication that the data are over-dispersed. In this case, a negative binomial model will be run. If the data are zero-inflated, then a zero-inflated Poisson or negative binomial model will be used. Incidence rate ratios and their associated 95% confidence intervals and *p* values will be provided.

The FTND, the Motivation to Quit questionnaire (MTQ), the Patient Health Questionnaire-9 (PHQ-9), the Generalised Anxiety Questionnaire-7 (GAD-7), the 12-item Short Form Health Survey (SF-12) physical component, SF-12 mental component scores, and BMI will all be analysed in the same way. Scores at 6 and 12 months will be compared between treatment groups using a repeated-measures covariance pattern mixed model including as fixed effects: baseline score, treatment group, time and a treatment group × time interaction term. Different covariance structures for the repeated measurements, that are available as part of STATA v13 (or later), will be explored and the most appropriate pattern will be used for the final model. Diagnostics including Akaike’s information criterion will be compared for each model (smaller values are preferred). Model assumptions will be checked. Estimates of the difference between treatment groups will be derived at all time points with 95% confidence intervals and *p* values.

Self-reported number of attempts to quit, periods of cessation, electronic cigarette use, and adverse events will be summarised descriptively by treatment group.

A complier average causal effect (CACE) analysis for the primary outcome will be conducted to obtain unbiased estimates of the intervention effectiveness in the presence of full compliance. We will define compliance with the intervention for those allocated to bespoke smoking cessation service (BSC) as the number of sessions attended.

### Economic analysis

Economic evaluation will be carried out in the form of an incremental cost-effectiveness analysis over the 12-month trial period, from a NHS and personal and social service perspective [18]. Intervention costs will be recorded by the research team and include delivery costs within the trial, supervision costs and appropriate capital costs. Resource use in physical units will be collected using an adapted Health Economic/Service Utilisation Questionnaire and case notes. Resource units will be valued by multiplying quantities by the corresponding market prices or national average unit costs.

Health-related quality of life will be measured using the EuroQol 5 dimensions, 5 levels (EQ-5D-5 L) questionnaire [19]. Health utilities are converted into quality-adjusted life years (QALYs), by calculating the area under curve [20]. An incremental cost-effectiveness ratio (ICER) will be calculated by combining difference in costs between groups and difference in QALYs between groups and compared with the national willingness-to-pay threshold to determine cost-effectiveness.

## Discussion

People with SMI experience significant health inequalities and one of the most important modifiable risk factors for reduced life expectancy is smoking. It is, therefore, important to develop evidence-based interventions to help people with SMI to quit smoking. The SCIMITAR+ trial is the largest trial to our knowledge to investigate the clinical effectiveness and cost-effectiveness of a bespoke smoking-cessation service for people with severe mental ill health.

### Study status

Recruitment to the SCIMITAR+ trial began in October 2015 and results are expected in 2018.

## Additional file

Additional file 1: Table S1. SPIRIT Checklist. (DOC 123 kb)

## Abbreviations

BMI: Body Mass Index
CMHT: Community Mental Health Team
CO: Carbon monoxide
DSM: *Diagnostic and Statistical Manual of Mental Disorders*
EQ-5D-5L: EuroQol 5 dimensions, 5 levels questionnaire
GAD: General anxiety disorder
GP: General practitioner
ICD: *International Classification of Diseases*
MH-SCP: Mental health smoking-cessation practitioner
NICE: National Institute of health and Care Excellence
NRT: Nicotine replacement therapy
PHQ: Patient Health Questionnaire
SCIMITAR: Smoking Cessation Intervention for severe Mental Ill health Trial
SF-12: 12-item Short Form Health Survey
SMI: Severe mental ill health

## References

[1] Brown S, Kim M, Mitchell C, Inskip H (2010). Twenty-five year mortality of a community cohort with schizophrenia. Br J Psychiatry.

[2] Jochelson K, Majrowski B (2006). Clearing the air: debating smoke-free policies in psychiatric units.

[3] Tam J, Warner K, Meza R. Smoking and the reduced life expectancy of individuals with serious mental illness. Am J Prev Med. In press. 10.1016/j.amepre.2016.06.007 27522471

[4] Cheeseman H, Harker K (2016). The stolen years.

[5] Myles N, Newall HD, Curtis J, Nielssen O, Shiers D, Large M (2012). Tobacco use before, at, and after first-episode psychosis: a systematic meta-analysis. J Clin Psychiatry.

[6] Williams JM, Ziedonis DM, Abanyie F (2005). Increased nicotine and cotinine levels in smokers with schizophrenia and schizoaffective disorder is not a metabolic effect. Schizophr Res.

[7] McCreadie R, Kelly C (2000). Patients with schizophrenia who smoke. Br J Psychiatry.

[8] McDonald C (2000). Cigarette smoking in patients with schizophrenia. Br J Psychiatry.

[9] Royal College of Physicians. Royal College of Psychiatrists Council Report CR178: smoking and mental health. Edited by RCP. London: Royal College of Physicians; 2013.

[10] Phelan M, Stradins L, Morrison S (2001). Physical health of people with severe mental illness: can be improved if primary care and mental health professionals pay attention to it. Br Med J.

[11] Ratschen E, Britton J, Doody GA, Leonardi-Bee J, McNeill A (2009). Tobacco dependence, treatment and smoke-free policies: a survey of mental health professionals’ knowledge and attitudes. Gen Hosp Psychiatry.

[12] Ratschen E, Britton J, Doody GA, McNeill A (2009). Smoke-free policy in acute mental health wards: avoiding the pitfalls. Gen Hosp Psychiatry.

[13] Lawn S, Feng Y, Tsourtos G, Campion J (2015). Mental health professionals’ perspectives on the implementation of smoke-free policies in psychiatric units across England. Int J Soc Psychiatry.

[14] Prochaska J (2011). Smoking and mental illness: breaking the link. N Engl J Med.

[15] Taylor G, McNeill A, Girling A, Farley A, Lindson-Hawley N, Aveyard P (2014). Change in mental health after smoking cessation: systematic review and meta-analysis. BMJ.

[16] Gilbody S, Peckham E, Man MS, Mitchell N, Li JS, Becque T, Hewitt C, Knowles S, Bradshaw T, Planner C (2015). Bespoke smoking cessation for people with severe mental ill health (SCIMITAR): a pilot randomised controlled trial. Lancet Psychiatry.

[17] Excellence NIfC (2013). Stop smoking services: public health guideline PH10.

[18] National Institute for Health and Care Excellence (NICE) (2013). Guide to the methods of technology appraisal 2013.

[19] The EuroQol Group (2013). EQ-5D-5L user guide: basic information on how to use the EQ-5D-5L instrument (version 2.0).

[20] Richardson G, Manca A (2004). Calculation of quality adjusted life years in the published literature: a review of methodology and transparency. Health Econ.

